# A Rare Case of Disseminated Histoplasmosis Involving the Colon and Brain

**DOI:** 10.7759/cureus.58046

**Published:** 2024-04-11

**Authors:** Nayna Nambiar, Gayathri S Hari, Priyanka Uppal

**Affiliations:** 1 Infectious Disease, Baylor Scott & White Medical Center, McKinney, USA; 2 Infectious Disease, Baylor Scott & White Medical Center, Mckinney, USA

**Keywords:** ring-enhancing brain lesion, colon ulcer, rectal ulcer, rare brain tumors, acute gi bleed, histoplamosis

## Abstract

*Histoplamsa capsulatum*, the causative organism for histoplasmosis, is a dimorphic fungus seen abundantly along the river valleys of Ohio and Mississippi in the United States of America as well as in other parts of the world. The infection is primarily acquired by inhaling the fungal spores which are often found in bird and bat droppings. Histoplasmosis can have a wide range of presentations ranging from no symptoms to mild flu-like or life-threatening consequences if severe. Chronic histoplasmosis can be akin to tuberculosis with a history of weight loss or hemoptysis. In patients with weak immune systems, histoplasmosis can become disseminated, affecting different parts of the body, which can be fatal if left untreated. We present a 40-year-old male with a past medical history of alcoholic cirrhosis and portal hypertension presenting with lower GI bleed found to have rectal and colonic ulcers as well as multiple brain lesions from disseminated histoplasmosis.

## Introduction

Histoplasmosis is an infection caused by a dimorphic fungus, *Histoplasma capsulatum*. This fungus has been found abundantly along the river valleys of Ohio and Mississippi in the United States of America. It is also seen in parts of Central and South America, Africa, Asia, and Australia. Infection is acquired primarily by inhalation of fungal spores, which are often found in bird and bat droppings. Histoplasmosis can have a wide range of presentations ranging from no symptoms to mild flu-like or life-threatening consequences if severe. Chronic histoplasmosis can sometimes mimic tuberculosis with a history of weight loss or hemoptysis [1,2]. However, in patients with weak immune systems, histoplasmosis can spread to different parts of the body, resulting in disseminated histoplasmosis, as seen in patients with acquired immune deficiency syndrome (AIDS) or leukemia, those on steroid therapy or chemotherapy, and recent transplant recipients. If left untreated, this can be fatal. Culture of organisms in blood, tissue, or other body samples remains the gold standard for diagnosing histoplasmosis. Other laboratory tests include imaging, antigen and antibody testing in body fluids and tissues, histopathological examination, and polymerase chain reaction, which can directly detect fungal DNA [3,4].

In this report, we present a case of disseminated histoplasmosis affecting the central nervous system and gastrointestinal tract in an immunocompromised patient from alcoholic cirrhosis.

## Case presentation

A 40-year-old Vietnamese male with a previous medical history of alcoholic cirrhosis with portal hypertension and esophageal varices, multiple upper gastrointestinal bleeds status post esophagogastroduodenoscopy (EGD) with banding twice in 2017 and 2021 presented with complaints of bright red blood per rectum. He also complained of decreased appetite, fatigue, and abdominal pain with distension. He had no history of non-steroidal anti-inflammatory drugs (NSAID) use.

He was admitted for management of acute lower gastrointestinal bleeding and a GI consultation was made. The patient was not able to give a much meaningful history, partly related to him being a poor historian from decreased mental status and due to a language barrier. Physical examination revealed anemia likely due to acute blood loss or splenic sequestration, a large palpable spleen most probably related to portal hypertension, and enlarged cervical lymph nodes. He underwent a CT abdomen pelvis with IV contrast, which showed a cirrhotic liver, splenomegaly, and portal hypertension.

He had underlying coagulopathy and raised bilirubin levels. The patient has not been drinking actively as per the history, but the aspartate transaminase (AST)/ alanine aminotransferase (ALT) pattern was concerning if he had used alcohol in the last few weeks or months. He underwent EGD, considering a prior history of esophageal varices to rule out upper GI bleed. The EGD revealed three columns of grade II varices in the lower third of the esophagus, red wale signs, and scarring from prior treatment. Three bands were successfully placed and there was a complete eradication of varices, which stopped the bleeding.

**Figure 1:**
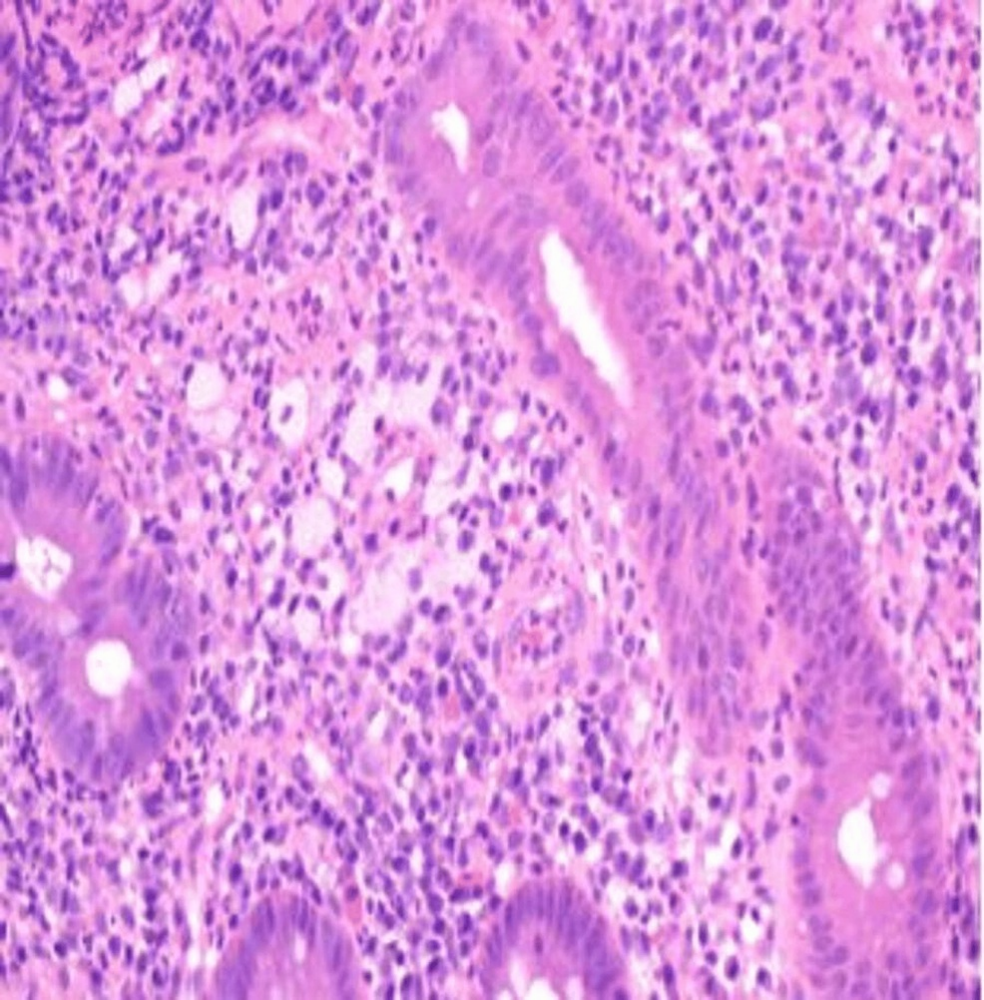
Histologic section of the colon showing acute colitis with numerous yeast forms within lamina propria histiocytes, 200x

**Figure 2:**
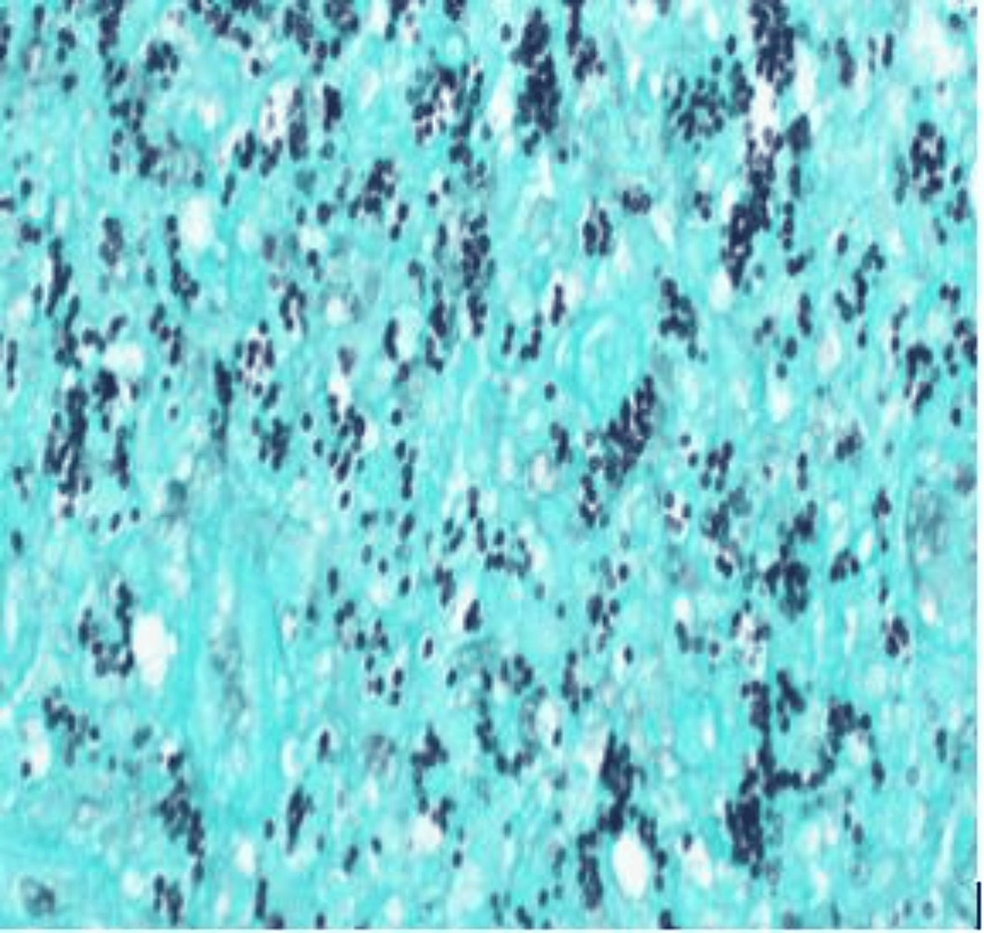
Positive GMS staining technique showing small uniform yeast cells characteristic of histoplasma, 400x GMS - Grocott's methenamine silver

The patient was empirically started on antibiotics and octreotide infusion for cirrhotic variceal bleeding. He continued to have rectal bleeding, and in the following day, he underwent a colonoscopy that showed significant ulceration (multiple ulcers 5mm-1cm) in the rectum, sigmoid colon, and descending colon; hemostatic clips were placed. Ulcers were biopsied. Rectal biopsy reported active colitis and was positive for histoplasmosis on Periodic acid-Schiff (PAS; Figure 1) and Grocott's methenamine silver (GMS) stain (Figure 2).

**Figure 3:**
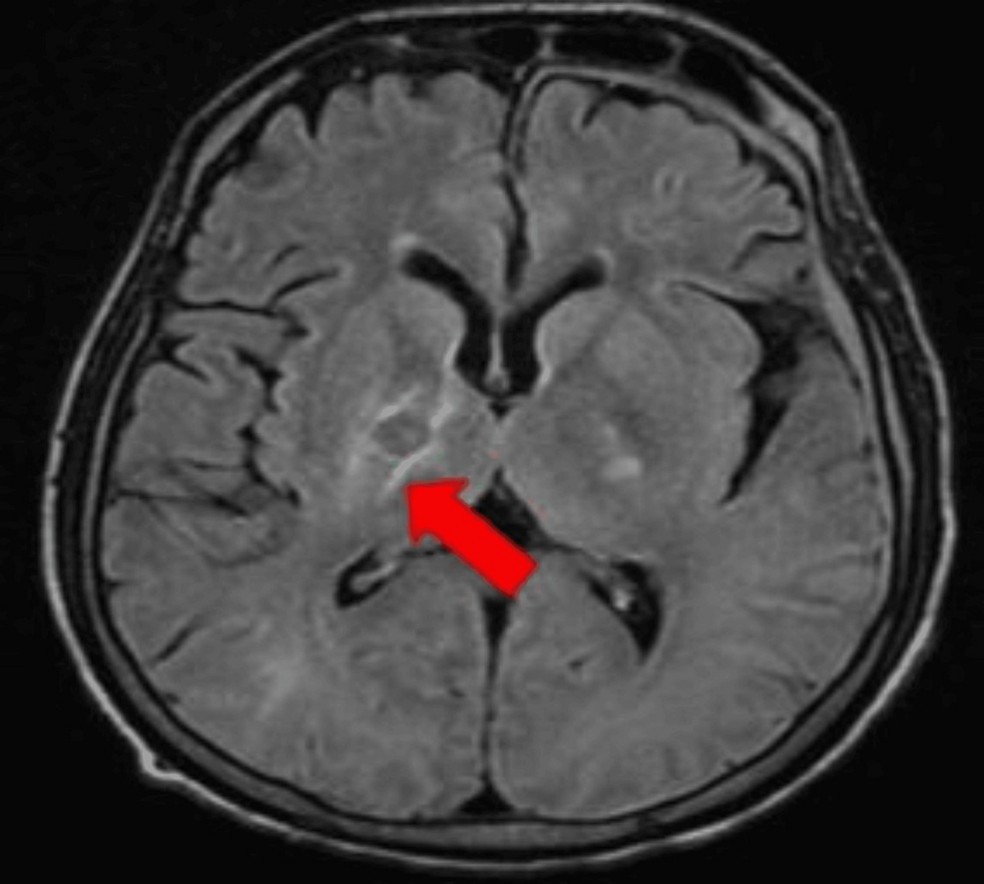
MRI brain illustrating ring-enhancing lesions as marked with an arrow

**Figure 4:**
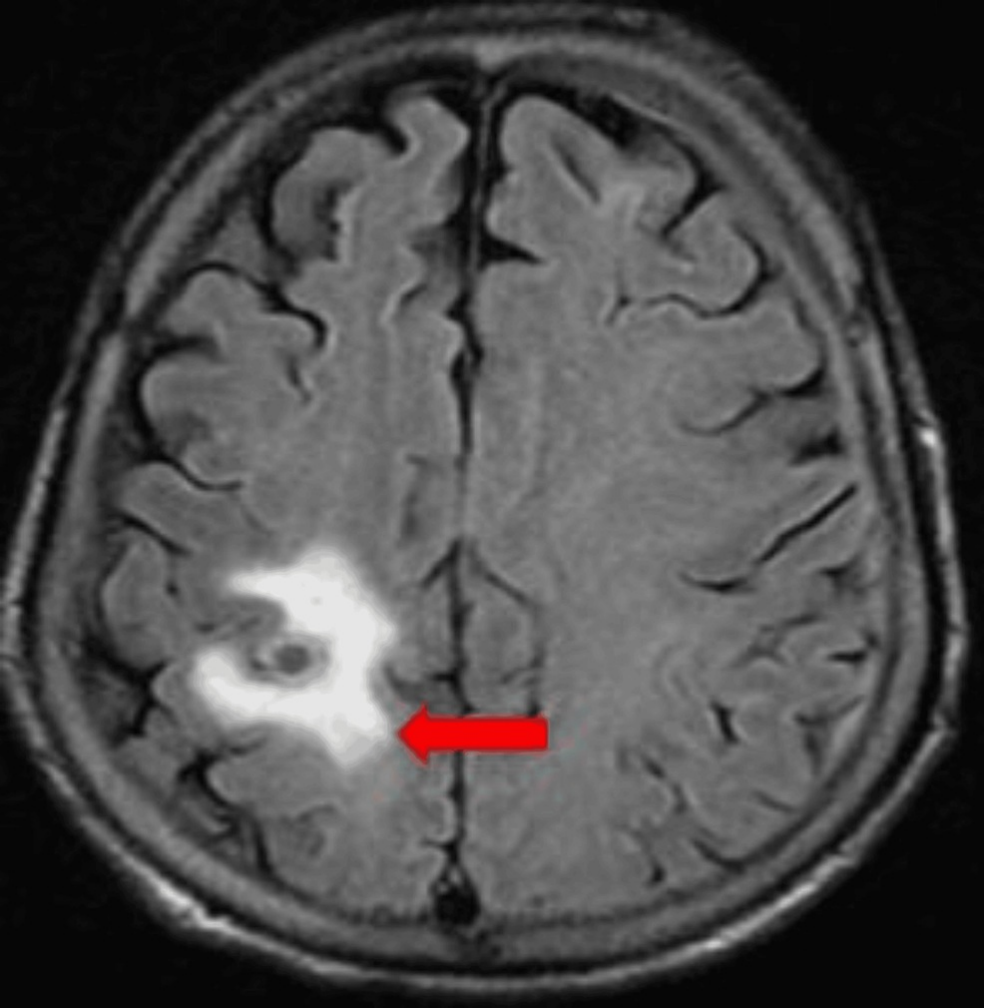
MRI brain illustrating ring-enhancing lesion as marked with an arrow

The patient underwent an MRI brain for altered mental status, which revealed multiple ring-enhancing lesions (Figures 3, 4). 

**Figure 5:**
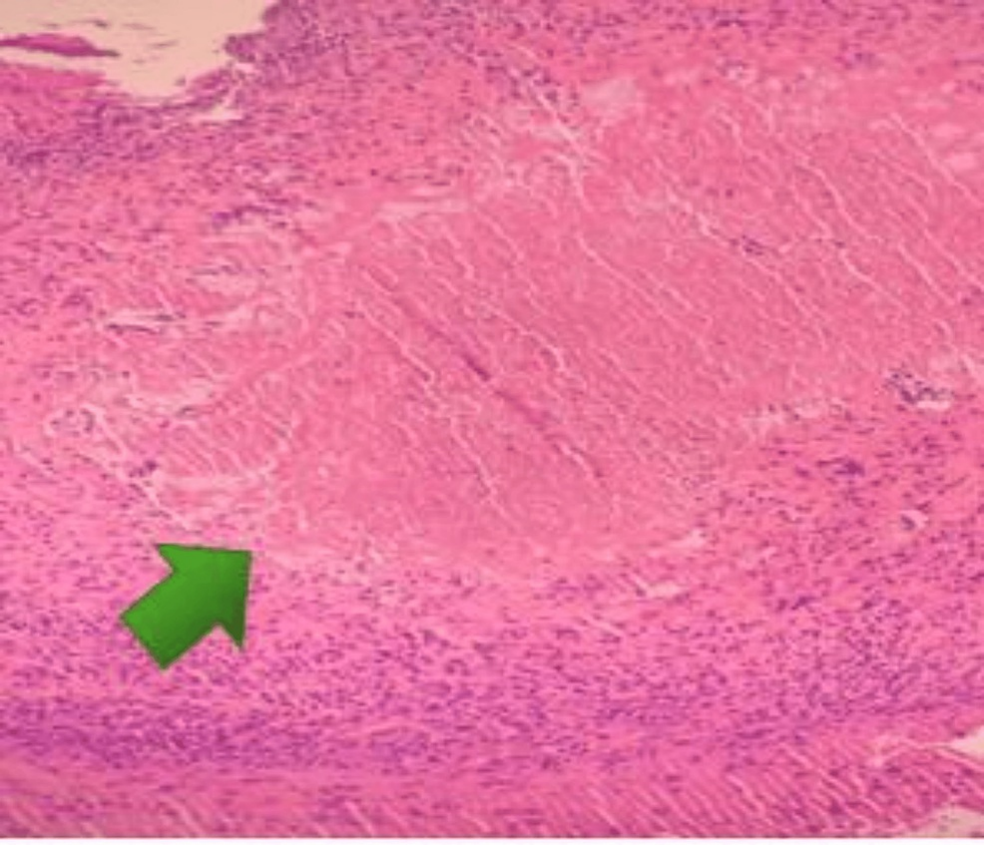
Histologic section of brain showing caseating granuloma with histoplasma, 100x

**Figure 6:**
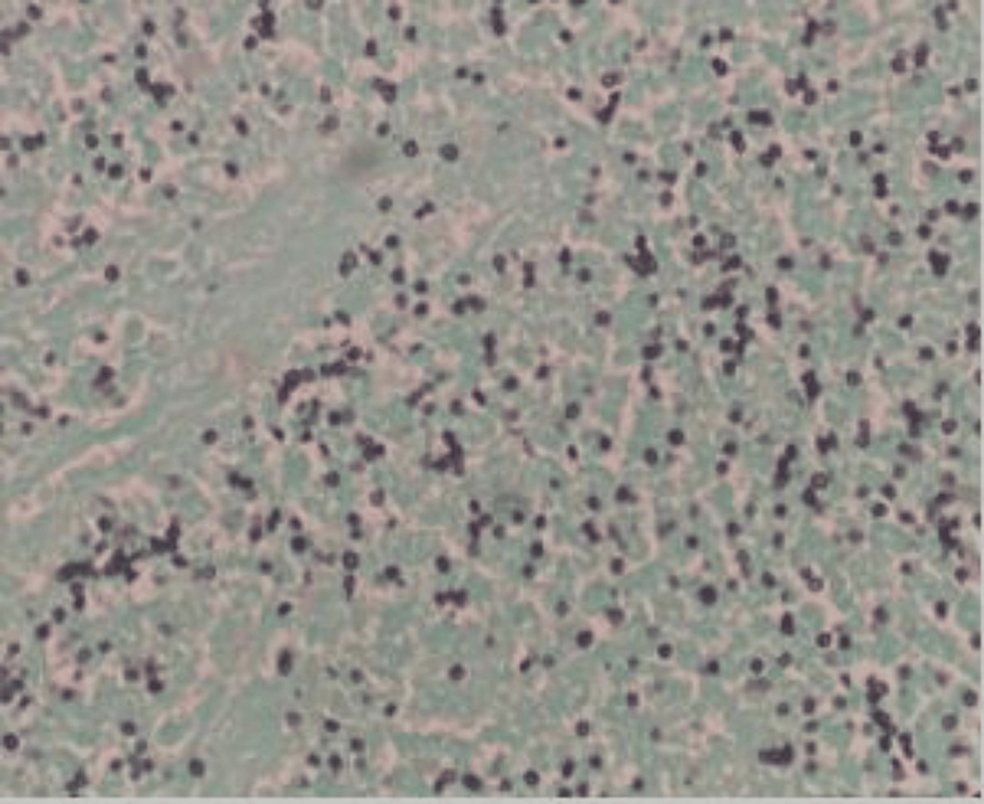
Brain tissue with positive GMS staining showing the presence of histoplasmosis, 200x GMS - Grocott's methenamine silver

CT chest was normal and infectious disease was consulted. The patient was started on intravenous amphotericin for treating disseminated histoplasmosis. Blood cultures were negative for fungal growth. His condition worsened, and he continued to have altered sensorium, rectal bleeding, and developed respiratory distress needing mechanical ventilation. A cerebrospinal fluid (CSF) study was negative for organisms. The patient underwent left temporal craniotomy and resection of the temporal mass, and the tissue biopsy revealed caseating granuloma with histoplasmosis on the PAS stain (Figure 5) and the presence of histoplasmosis on the GMS stain (Figure 6).

He remained unresponsive and continued to bleed in the brain in follow-up scans. Despite all treatments, he expired from multi-organ failure.

## Discussion

*Histoplasma capsulatum* (*H. capsulatum*) is a fungus that causes histoplasmosis - a flu-like illness with mild pulmonary symptoms. This fungus grows in soil and has been isolated from the feces of birds in endemic areas. In the majority of patients, the infection is self-limited and localized, affecting only the lungs, the cervical lymph nodes, or both. However, in patients with a weak immune system, histoplasmosis can spread to different parts of the body, resulting in disseminated histoplasmosis. Widespread infection is associated with 100% mortality if untreated or improperly treated. Even in patients treated with IV amphotericin B and ketoconazole, mortality is about 50% [1-3].

Gastrointestinal lesions occur in nearly 75% of patients with disseminated histoplasmosis [2-4]. Gastrointestinal histoplasmosis (GIH), though predominant in the immunocompromised, can also be found in immunocompetent individuals. GIH mimics several gastrointestinal conditions. So, despite the increasing incidence of disseminated histoplasmosis (DH), GIH is rarely reported. Lack of data on GIH may also be due to neglect of the fungal disease, lack of awareness or adequate diagnostic procedures such as colonoscopy. 

The signs and symptoms of GIH can be very nonspecific. It can present as abdominal pain, diarrhea, fever, weight loss, vomiting, constipation, hematemesis and hematochezia. The presentations can often be misdiagnosed as malignancy or acute exacerbation of inflammatory bowel disease, especially in patients already on immunosuppressants or tuberculosis. Lesions of GIH are usually seen at the terminal ileum, ileocecal junction, and ascending colon, presumably due to the abundance of lymphoid tissue in these areas. The initial focal lesions may be small, patchy superficial ulcers and raised plaques with surrounding granular and erythematous mucosa. Associated endoscopic findings can range from intestinal ulcerations, strictures, and friable masses to ulcerated polyps [5-8].

Usual complications of unattended GH include lower gastrointestinal bleeding, intestinal perforation, ulceration, intestinal obstruction, acute pancreatitis, acute cholecystitis, and death [7-13].

The central nervous system is involved in about 5 to 20% of cases of disseminated histoplasmosis. It is more common in those with underlying immunosuppressive disorders [6]. Disseminated histoplasmosis (DH), with a ring-enhancing brain lesion, is not usually seen in an immunocompetent individual. Among those who develop DH in the central nervous system (CNS), nearly 25% will develop neurological symptoms. Brain imaging may show hydrocephalus, histoplasma, vasculitis with infarctions, and leptomeningeal enhancement [4,14-17]. One characteristic feature of fungal etiology is the projections into the cavity from the wall of an abscess with a low apparent diffusion coefficient and no enhancement. Pathological studies of brain tissue may show organisms.

The strongest historical evidence of infection is the isolation of *H. capsulatum*, but it may take up to four weeks. Blood and bone marrow are the most likely specimens to yield *H. capsulatum* in DH. Conventional blood culture, lysis-centrifugation (isolator), and glycoprotein antigen detection in blood or urine specimens can be useful in diagnosing GI histoplasmosis. Pathological examination and fungal culture of GI tissue specimens are other sensitive methods for diagnosing GI histoplasmosis [8].

The mainstay of therapy in DH is the use of systemic antifungals; amphotericin B is used for induction therapy and is found to be superior to itraconazole. Itraconazole can be used to treat mild cases of DH and is effective in preventing relapses [11,12]. It may be used for maintenance therapy owing to the ease of administration and lesser side effects, with attention paid to potential drug interactions. Treatment with fluconazole in preventing relapses may be considered for individuals who require rifampin or in whom there is poor absorption of itraconazole, but failures have been reported. However, relapses can occur despite maintenance therapy.

DH is associated with high mortality, and withholding the treatment can cause rapid death. Surgical intervention may be indicated in cases of bowel perforation and refractory bowel obstruction due to GI histoplasmosis, but no general recommendations can be made. However, it is unclear whether surgical resection, in addition to systemic antifungal therapy, might lead to an improved outcome.

## Conclusions

This case report provides a unique contribution to the literature by documenting a rare case of disseminated histoplasmosis involving the gastrointestinal tract and nervous system in an immunocompromised individual. Our findings underscore the importance of comprehensive evaluation and differential diagnosis in the management of gastrointestinal ulcers and ring-enhancing brain lesions. Continued study is necessary to understand the potential causes, preventive measures, and treatment of such conditions.
